# Case Report: Life-saving external hemipelvectomy after missed external iliac artery occlusion in severe pelvic trauma

**DOI:** 10.3389/fsurg.2026.1878507

**Published:** 2026-07-24

**Authors:** Nian-Jhen Wu, Dun-Hao Chang, Yu-Hung Chen

**Affiliations:** 1 Department of Orthopedic Surgery, Far-Eastern Memorial Hospital, New Taipei City, Taiwan; 2 Department of Plastic & Aesthetic Surgery, Far Eastern Memorial Hospital, New Taipei City, Taiwan

**Keywords:** deep inferior epigastric perforator flap, external hemipelvectomy, limb ischemia, pelvic trauma, traumatic external iliac artery occlusion

## Abstract

External hemipelvectomy is a radical procedure traditionally reserved for advanced malignancy, with its use in trauma being exceedingly rare. We report a 15-year-old female with severe pelvic trauma complicated by a missed external iliac artery occlusion following internal iliac artery embolization, resulting in irreversible limb ischemia and progressive soft-tissue necrosis. Despite fasciotomy, above-knee amputation, and repeated debridement, the infection advanced proximally with recurrent sepsis, ultimately necessitating a life-saving external hemipelvectomy. Reconstruction was achieved using a deep inferior epigastric artery perforator flap, followed by staged wound management and rehabilitation, leading to complete wound healing. At 28 months of follow-up, the patient was able to ambulate independently with a customized hemipelvic prosthesis. This case highlights a critical diagnostic pitfall in pelvic trauma and demonstrates that timely recognition, multidisciplinary management, and advanced reconstructive strategies can achieve meaningful functional recovery even after radical limb-sacrificing procedures.

## Introduction

External hemipelvectomy is a radical, limb-sacrificing procedure traditionally reserved for advanced malignancy, uncontrollable infection, or catastrophic musculoskeletal destruction when limb salvage is no longer feasible (1–4). Its application in trauma settings is exceedingly rare (5), and typically represents a last-resort, life-saving intervention (4, 6).

In trauma settings, high-energy pelvic injuries may involve multisystem compromise, complex musculoskeletal disruption, and major vascular injury (7, 8). However, arterial injuries may be overlooked during initial resuscitation when hemodynamic instability limits comprehensive assessment (9, 10). Missed external iliac artery occlusion can rapidly progress to irreversible limb ischemia, extensive soft-tissue necrosis, and systemic deterioration (7, 11, 12), ultimately forcing the need for external hemipelvectomy as the only viable life-saving option (5, 13, 14).

Given the rarity of trauma-related external hemipelvectomy, its indications, surgical considerations, and functional outcomes remain poorly characterized. This case report describes a young patient who underwent life-saving external hemipelvectomy following missed external iliac artery occlusion after pelvic trauma and highlights a critical diagnostic pitfall as well as the potential for meaningful functional recovery through multidisciplinary management.

## Case presentation

**Figure 1:**
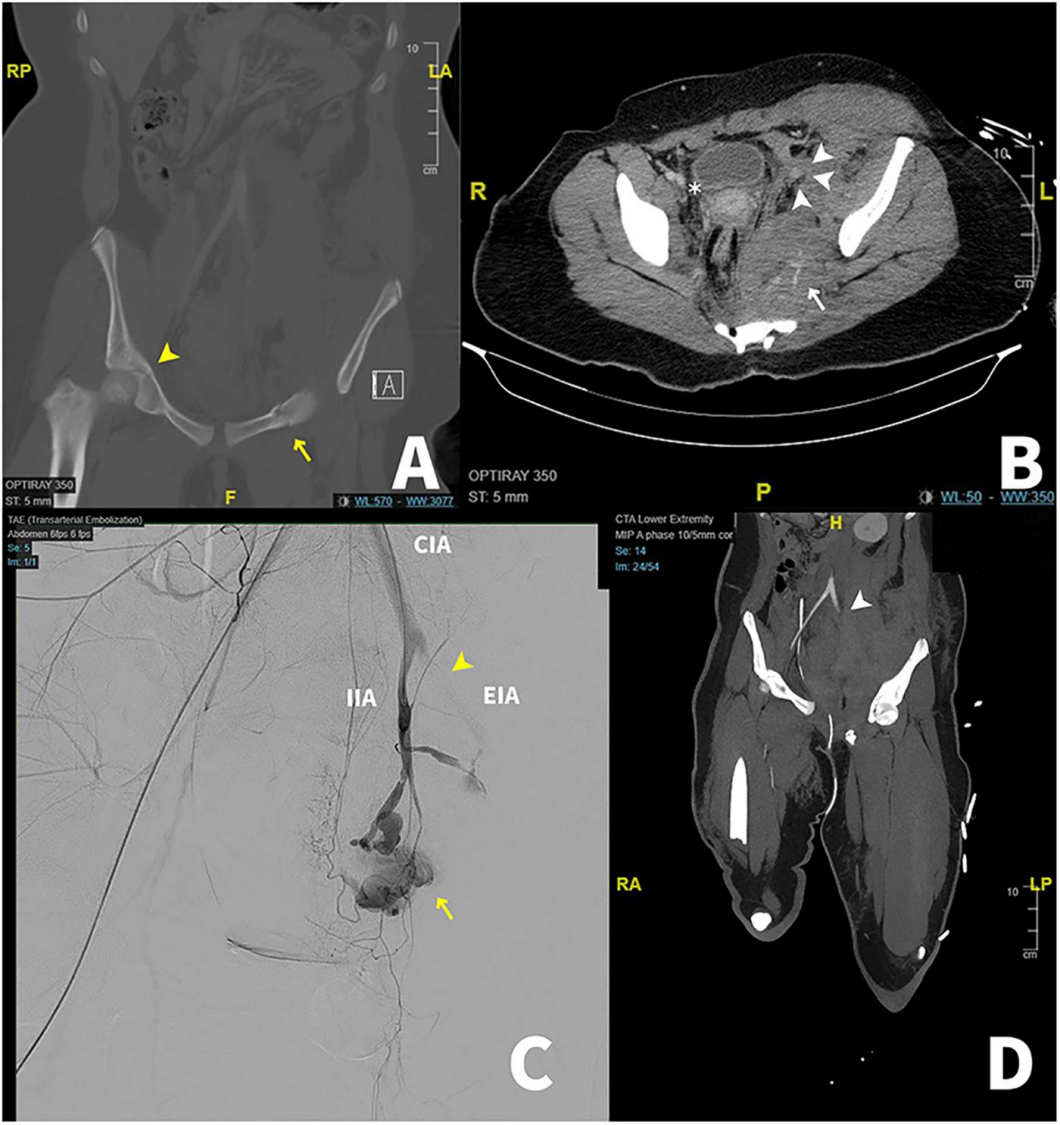
Initial contrast-enhanced computed tomography (CT) scan **(A,B)**, transarterial embolization and subsequent emergent computed tomography angiography **(C,D)**. **(A)** Bone window of the scan. The yellow arrowhead indicates a left pubic ramus fracture. The yellow arrow indicates a right acetabular fracture. **(B)** The white arrow indicates contrast extravasation from the left pelvic wall. The white asterisk marks the normal right external iliac artery and vein. The white arrowheads indicate a compromised left external iliac artery. **(C)** Angiography showing labeled vessels: common iliac artery (CIA), IIA and EIA. The yellow arrow indicates contrast extravasation. The yellow arrowhead indicates the initially neglected obstructed left external iliac artery. **(D)** The white arrowhead indicates a completely obstructed left external iliac artery.

A 15-year-old, otherwise healthy female passenger on a motorcycle was involved in a collision with a car and was thrown onto another vehicle's roof. On arrival of the emergency room, she presented in profound hypovolemic shock with a heart rate of 158 bpm, unmeasurable blood pressure, and tachypnea (respiratory rate 30 breaths/min). Her Glasgow Coma Scale (GCS) was 9 (E2V2M5). Massive transfusion protocol was immediately initiated for resuscitation. Whole-body computed tomography (CT) scan revealed complex pelvic fractures involving the left sacral ala, right acetabulum, and bilateral ischium (Figure 1A), accompanied by pubic symphysis diastasis and a large retroperitoneal hematoma. There was contrast extravasation along the left pelvic sidewall, consistent with active arterial bleeding (Figure 1B). Retrospective review of the initial trauma CT angiography demonstrated complete occlusion of the left external iliac artery. However, this finding was not recognized during the initial assessment because clinical attention was primarily directed toward hemorrhage control and management of hemodynamic instability. Although gross limb perfusion was not initially recognized as compromised, a detailed vascular examination of the left lower extremity, including pulse assessment and Doppler ultrasonography, was not fully documented during the initial assessment.

The patient underwent emergent angiography, which confirmed hemorrhage from the left internal iliac artery (IIA). Transcatheter arterial embolization (TAE) of the left IIA was successfully performed to stabilize hemodynamics (Figure 1C), and she was transferred to the Intensive Care Unit (ICU) for postoperative monitoring. However, during reassessment approximately 8 h after the initial trauma CT examination, the patient's left lower extremity was noted to be cold and pulseless. Emergent CT angiography demonstrated complete occlusion of the left external iliac artery (EIA) (Figure 1D). A cardiovascular surgeon was consulted, and the estimated warm ischemia time was approximately 8.5 h, calculated from the time of the initial trauma CT examination to the time of cardiovascular surgical consultation as a conservative estimation. Given the prolonged ischemic duration and clinical evidence of irreversible limb ischemia, the limb was retrospectively classified as Rutherford category III acute limb ischemia, and revascularization was considered contraindicated. Surgical thrombectomy and bypass reconstruction were discussed but ultimately not pursued because restoration of blood flow was considered unlikely to salvage the limb and carried a substantial risk of severe reperfusion syndrome, potentially resulting in life-threatening systemic complications.

Despite aggressive supportive care, the patient developed rapidly progressive ischemic necrosis accompanied by severe sepsis, manifested by persistent hypotension, tachycardia, fever, lactic acidosis, leukocytosis and bandemia. Even fasciotomy and above-knee amputation couldn't avoid ischemic and infectious process extending proximally, leading to recurrent sepsis. A damage-control hip disarticulation was subsequently performed but failed to achieve viable margins. Given the extensive ischemic necrosis, uncontrolled necrotizing infection, and ongoing hemodynamic instability, definitive source control was considered necessary. After multidisciplinary discussion and detailed communication with the patient's family regarding the patient's young age and the possibility of meaningful functional recovery, aggressive treatment was pursued, and a life-saving left external hemipelvectomy was ultimately performed on post-injury day (PID) 24. Intraoperatively, extensive necrosis of the pelvic soft tissues was noted, with involvement of the gluteal maximus, gluteal medius, sciatic nerve, and lumbar nerve roots. A large purulent collection extending from the perineal region to the paraspinal space was also identified. The affected hemipelvis was resected *en bloc* (Supplementary Figure 1), and the left common iliac vessels were ligated. The wound was packed with wet dressings and left open for further wound management.

**Figure 2:**
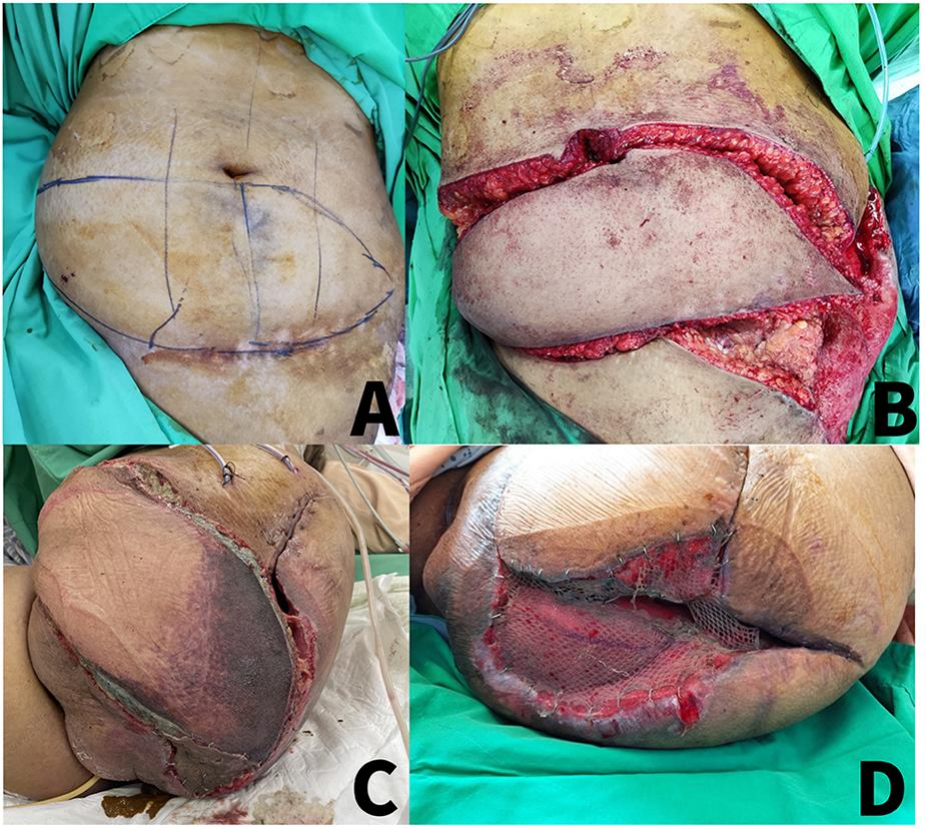
Reconstruction of the wound of external hemipelvectomy. **(A)** Preoperative skin marking for flap design. Left periumbilical perforator (red dot) was used as pivot point with counter-clockwise propeller rotation (Supplementary Video 1). The white arrows indicate the direction of flap rotation. **(B)** Pedicled DIEP propeller flap after rotation. **(C)** Partial necrosis of the distal portion of the flap. **(D)** Wound condition after local debridement and split-thickness skin grafting.

After hemipelvectomy, the wound was cared by serial debridement and negative pressure wound therapy with continuous irrigation. After the infection subsided, plastic surgeon performed extensive debridement and transposition of a pedicled deep inferior epigastric artery perforator (DIEP) propeller flap on PID 59 for pelvic wound coverage. Preoperative CTA perforator mapping and intraoperative hand-held Doppler ultrasonography was used to assess perforator patency and identify three suitable left periumbilical perforators, and the largest one was chosen to faciliate the propeller rotation. A pedicled left DIEP flap measuring approximately 35 × 10 cm, based on the left rectus abdominis vascular territory, was elevated and rotated counterclockwise by approximately 120° to cover the pelvic defect while preserving pedicle perfusion. However, partial necrosis of the distal flap (at Hartrampf's Zones of Perfusion IV) developed, and it was successfully managed through local debridement and split-thickness skin grafts (Figure 2, Supplementary Video 1).

## Follow-up and outcomes

**Figure 3:**
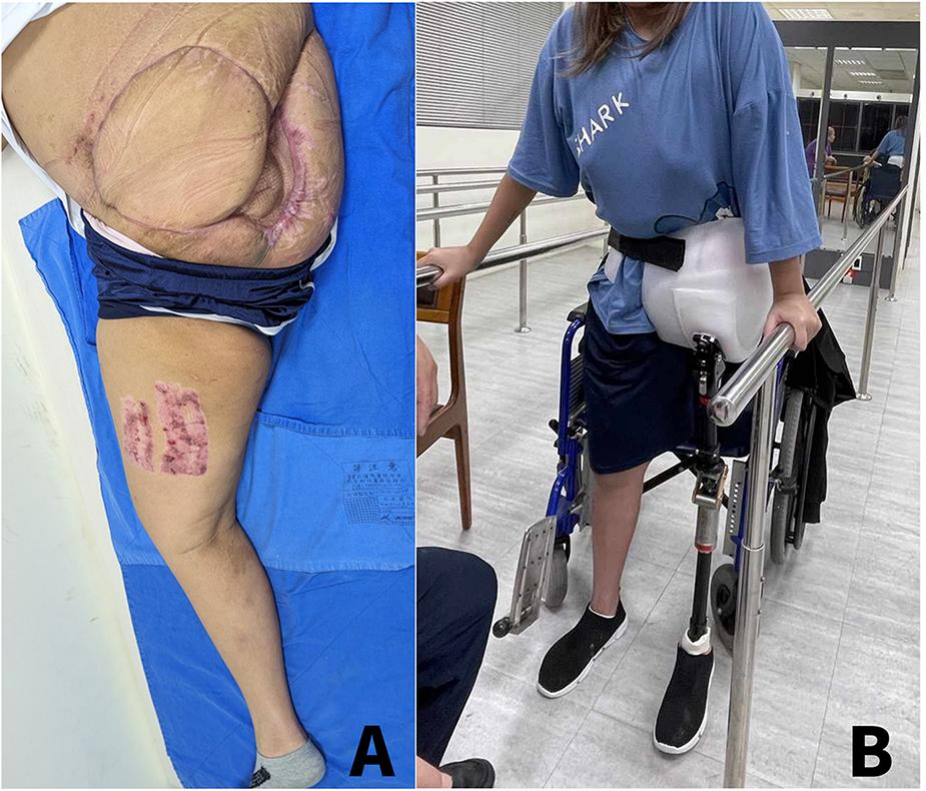
Wound condition at discharge and subsequent rehabilitation. **(A)** Complete epithelialization of the wound at the time of discharge. **(B)** Functional rehabilitation with a customized hemipelvic prosthesis (Supplementary Video 2).

By PID 131, the pelvic wound had achieved complete epithelialization, and the patient was discharged. She subsequently underwent a comprehensive rehabilitation program focusing on core strengthening and prosthetic training. At the latest outpatient follow-up (post-injury 28 months), the patient demonstrated independent, full weight-bearing ambulation using a customized hemipelvic prosthesis, requiring no assistive devices (Figure 3, Supplementary Video 2). The clinical progression and therapeutic interventions are summarized in the timeline presented in Table 1.

**Table 1:** Timeline of clinical events

Time	Event
PID 0	Traffic accident with pelvic fracture and retroperitoneal bleeding.
PID 0, 3 h after initial CT scan	TAE to IIA
PID 0, 8 h after initial CT scan	Left lower limb pulseless and cold. CTA found completely obstructed left external iliac artery
PID 0, 8.5 h after initial CT scan	Cardiovascular surgeon consultation, revascularization not pursued. Emergent fasciotomy
PID 3	Above-knee amputation
PID 5	Stump revision and debridement
PID 8	Stump revision and debridement
PID 10	Stump revision and debridement
PID 12	Stump revision and debridement
PID 15	Disarticulation of left hip
PID 24	External hemipelvectomy
PID 30	Debridement
PID 33	Debridement
PID 39	Debridement
PID 44	Debridement
PID 59	Debridement with pedicled DIEP propeller flap
PID 64	Partial necrosis of the distal portion of the flap, debridement
PID 72	Debridement
PID 80	Debridement
PID 92	Split-thickness skin graft for wound coverage
PID 131	Discharge

## Discussion

External hemipelvectomy is an uncommon and radical operation performed predominantly in the management of advanced pelvic malignancies when limb preservation is no longer feasible (4, 15). Its use in trauma is exceedingly rare, and only isolated reports describe its role as a life-saving procedure in severe musculoskeletal destruction or overwhelming infection (5, 13). External hemipelvectomy is associated with considerable morbidity and mortality. Reported perioperative mortality rates (in-hospital) range from approximately 5% to 8.8% in some series (4, 16–18), underscoring the severe underlying conditions that typically necessitate this operation.

The reported incidence of missed injuries in trauma patients ranges widely from 0.6% to 39% (19). Vascular injuries remain challenging to detect during early resuscitation, particularly in profoundly unstable patients (19, 20). In such situations, priority is directed toward hemorrhage control, and distal perfusion may not be immediately reassessed. External iliac artery occlusion is uncommon and can be easily overlooked (10, 14, 21), especially when initial interventions temporarily stabilize hemodynamics. However, delayed recognition can lead to irreversible ischemia, rapid extension of soft-tissue necrosis, and systemic deterioration (14, 21). In our case, the external iliac artery occlusion was likely caused by traumatic thrombosis or intimal injury associated with pelvic ring disruption. While the pelvic collateral circulation is generally robust (22, 23), the combination of internal iliac artery embolization and external iliac artery occlusion created a watershed area of profound ischemia involving the gluteal muscles and the hip joint envelope. In this situation, hemipelvectomy became the only viable option to achieve surgical margins with bleeding, viable tissue (14).

Reconstruction following external hemipelvectomy poses significant challenges due to the magnitude of soft-tissue defects and high risk of contamination (2, 18, 24, 25). Unlike in elective tumor resections, a major reconstructive challenge in trauma cases is the unavailability of the “fillet flap” from the lower extremity (24–28), as the limb is often already compromised, as seen in this patient. Given the complete destruction of the ipsilateral external and internal iliac arteries, the local vascular environment was severely compromised, leaving no suitable recipient vessels for microvascular anastomosis. As a result, conventional free flap reconstruction was not feasible. Instead, a pedicled DIEP propeller flap was utilized, which allowed soft-tissue coverage without the need for microvascular anastomosis to the damaged pelvic vessels, thereby effectively bypassing the vascular limitation.

Although perfusion of the left deep inferior epigastric artery (DIEA) would be expected to be compromised due to external iliac artery occlusion, collateral flow from the superior epigastric artery may have helped maintain perfusion (29). This is also the reason we used propeller design and did not completely skeletonized the DIEA. This decision was critical not only for ensuring flap survival in a devascularized bed but also for providing the durable, bulky soft-tissue envelope required for prosthetic fitting (30). According to Hartrampf's perfusion zones, Zone IV represents the region farthest from the dominant perforators and is therefore most vulnerable to ischemia (31, 32). In this case, flap survival was likely supported by collateral retrograde inflow through the superior epigastric circulation despite complete interruption of the ipsilateral iliac inflow. The distal flap necrosis observed in Zone IV further supports this vascular pattern.

Despite the successful life-saving outcome and functional recovery observed in this case, several limitations must be acknowledged. As a single-patient case report, the clinical findings and the specific success of the pedicled DIEP propeller flap reconstruction may not be directly generalizable to all patients. Furthermore, although the patient achieved independent ambulation with a prosthesis at the latest follow-up, the long-term durability of the soft tissue reconstruction and the potential for late-onset prosthetic complications require extended clinical observation. Finally, the specific watershed ischemia caused by the combination of internal iliac artery embolization and neglected external iliac artery occlusion represents a rare and complex clinical scenario, which may limit the applicability of this specific surgical strategy to patients with different patterns of vascular injury.

Historically, a significant proportion of patients undergoing external hemipelvectomy remain permanently wheelchair-dependent for daily mobility (6, 16). However, this case illustrates that independent ambulation remains an attainable goal. The durable soft tissue coverage provided by the reconstruction created a stable interface for the prosthesis, allowing the patient to regain full weight-bearing capacity. This confirms that while hemipelvectomy is a radical procedure, effective reconstruction combined with rehabilitation can still yield an acceptable functional outcome.

## Patient perspective

The patient reported that although the initial injury and subsequent surgeries were physically and emotionally challenging, she gradually adapted to her condition through rehabilitation. She expressed satisfaction with the surgical outcome, particularly her ability to regain independent ambulation with a prosthesis.

## Conclusion

External hemipelvectomy serves as a formidable but necessary life-saving intervention when damage control amputation fails to arrest progressive sepsis. This case highlights a critical diagnostic pitfall in pelvic trauma: the combination of internal iliac artery embolization and an unrecognized external iliac artery occlusion may create a profound pelvic ischemic zone. Ultimately, while hemipelvectomy is a radical procedure often associated with permanent disability, this report demonstrates that decisive surgical management followed by robust orthoplastic reconstruction can provide a durable stump. Even in such catastrophic scenarios, an acceptable functional outcome with independent ambulation remains an attainable goal.

## Data Availability

The original contributions presented in the study are included in the article/Supplementary Material, further inquiries can be directed to the corresponding author.

## Supplementary material

Supplementary Figure 1 The resected hemipelvis and necrotic tissue.

Supplementary Video 1 Intraoperative demonstration of pedicled deep inferior epigastric artery perforator (DIEP) propeller flap rotation and inset for coverage of the left pelvic defect.

Supplementary Video 2 Post-rehabilitation functional outcome showing stair ambulation using a customized hemipelvic prosthesis.

## References

[1] Karakousis CP Emrich LJ Driscoll DL. Variants of hemipelvectomy and their complications. Am J Surg. (1989) 158(5):404–8. 10.1016/0002-9610(89)90273-0 2817220

[2] Karaca MO Ozbek EA Ozyildiran M Merter A Basarir K Yildiz HY. External and internal hemipelvectomy: a retrospective analysis of 68 cases. Jt Dis Relat Surg. (2022) 33(1):132–41. 10.52312/jdrs.2022.560 35361087 PMC9057530

[3] Mayerson JL Wooldridge AN Scharschmidt TJ. Pelvic resection: current concepts. J Am Acad Orthop Surg. (2014) 22(4):214–22. 10.5435/JAAOS-22-04-214 24668351

[4] Guder WK Hardes J Gosheger G Henrichs MP Nottrott M Streitburger A. Analysis of surgical and oncological outcome in internal and external hemipelvectomy in 34 patients above the age of 65 years at a mean follow-up of 56 months. BMC Musculoskelet Disord. (2015) 16:33. 10.1186/s12891-015-0494-5 25888345 PMC4342034

[5] Kołodziejski L Duber J Pomykacz P Komorowski AL. External hemipelvectomy: a last resort operation. Nowotwory Journal of Oncology. (2023) 73(2):81–4. 10.5603/NJO.a2023.0014

[6] Ramos Pascua LR Casas Ramos P De la Cruz Gutierrez L Negri ME Vilar Gonzalez E Cordova Peralta JC. External hemipelvectomy in soft tissue sarcomas: are they still needed? Cancers (Basel). (2024) 16(22):3828. 10.3390/cancers16223828 39594784 PMC11593110

[7] Aoki R Nakajima K Kobayashi Y Sakai Y Kamide H Yamamoto T. Common and uncommon vascular injuries and endovascular treatment associated with pelvic blunt trauma: a real-world experience. Jpn J Radiol. (2023) 41(3):258–65. 10.1007/s11604-022-01355-1 36350523 PMC9974705

[8] Coccolini F Stahel PF Montori G Biffl W Horer TM Catena F. Pelvic trauma: wSES classification and guidelines. World J Emerg Surg. (2017) 12:5. 10.1186/s13017-017-0117-6 28115984 PMC5241998

[9] Harris DG Drucker CB Brenner ML Narayan M Sarkar R Scalea TM. Management and outcomes of blunt common and external iliac arterial injuries. J Vasc Surg. (2014) 59(1):180–5. 10.1016/j.jvs.2013.07.107 24140115

[10] Zhang S Sheng H Xu B Lao Y. Acute external iliac artery thrombosis following pelvic fractures: two case reports. Medicine (Baltimore). (2021) 100(6):e24710. 10.1097/MD.0000000000024710 33578610 PMC10545012

[11] de Castro-Santos G. The semantics of acute limb ischemia. J Vasc Bras. (2023) 22:e20210145. 10.1590/1677-5449.202101451 38379534 PMC10878674

[12] Galyfos G Chamzin A Intzes N Matthaiou G Spiliotopoulos S Sotirakis D. Acute limb ischemia. J Cardiovasc Surg (Torino). (2023) 64(4):396–405. 10.23736/S0021-9509.22.12536-X 36762508

[13] Hernandez J Rosenthal AA. Hemipelvectomy as a salvage procedure in massive pelvic trauma. Am Surg. (2022) 88(7):1568–9. 10.1177/00031348221084939 35465736

[14] Pascarella R Del Torto M Politano R Commessatti M Fantasia R Maresca A. Critical review of pelvic fractures associated with external iliac artery lesion: a series of six cases. Injury. (2014) 45(2):374–8. 10.1016/j.injury.2013.10.011 24183394

[15] Banskota N Yang H Fang X Yuan D Zhang W Duan H. Comparative study of pelvic sarcoma patients undergoing internal and external hemipelvectomy: a meta-analysis study. Front Surg. (2022) 9:988331. 10.3389/fsurg.2022.988331 36311928 PMC9614061

[16] Apffelstaedt JP Driscoll DL Spellman JE Velez AF Gibbs JF Karakousis CP. Complications and outcome of external hemipelvectomy in the management of pelvic tumors. Ann Surg Oncol. (1996) 3(3):304–9. 10.1007/BF02306287 8726187

[17] Apffelstaedt JP Zhang PJ Driscoll DL Karakousis CP. Various types of hemipelvectomy for soft tissue sarcomas: complications, survival and prognostic factors. Surg Oncol. (1995) 4(4):217–22. 10.1016/s0960-7404(10)80038-6 8528484

[18] Senchenkov A Moran SL Petty PM Knoetgen J Clay RP Bite U. Predictors of complications and outcomes of external hemipelvectomy wounds: account of 160 consecutive cases. Ann Surg Oncol. (2008) 15(1):355–63. 10.1245/s10434-007-9672-5 17955297

[19] Pfeifer R Pape HC. Missed injuries in trauma patients: a literature review. Patient Saf Surg. (2008) 2:20. 10.1186/1754-9493-2-20 18721480 PMC2553050

[20] Perry MO. Complications of missed arterial injuries. J Vasc Surg. (1993) 17(2):399–407. 8433434

[21] Ahn J Kim JW. External iliac artery injury with posterior pelvic ring injury in Korea: two case reports. J Trauma Inj. (2023) 36(2):137–41. 10.20408/jti.2022.0046 39380705 PMC11309450

[22] Chait A Moltz A Nelson JH Jr. The collateral arterial circulation in the pelvis. An angiographic study. Am J Roentgenol Radium Ther Nucl Med. (1968) 102(2):392–400. 10.2214/ajr.102.2.392 5635691

[23] Usui R Kondo H. Transcatheter arterial embolization for hemorrhagic pelvic fracture: review article. Interv Radiol (Higashimatsuyama). (2024) 9(3):156–63. 10.22575/interventionalradiology.2023-0015 39559807 PMC11570184

[24] Mat Saad AZ Halim AS Faisham WI Azman WS Zulmi W. Soft tissue reconstruction following hemipelvectomy: eight-year experience and literature review. ScientificWorldJournal. (2012) 2012:702904. 10.1100/2012/702904 22629187 PMC3353558

[25] Senchenkov A Moran SL Petty PM Knoetgen J Tran NV Clay RP. Soft-tissue reconstruction of external hemipelvectomy defects. Plast Reconstr Surg. (2009) 124(1):144–55. 10.1097/PRS.0b013e3181a80557 19568053

[26] Kuntscher MV Erdmann D Homann HH Steinau HU Levin SL Germann G. The concept of fillet flaps: classification, indications, and analysis of their clinical value. Plast Reconstr Surg. (2001) 108(4):885–96. 10.1097/00006534-200109150-00011 11547143

[27] Kreutz-Rodrigues L Mohan AT Moran SL Carlsen BT Mardini S Houdek MT. Extremity free fillet flap for reconstruction of massive oncologic resection-surgical technique and outcomes. J Surg Oncol. (2020) 121(3):465–73. 10.1002/jso.25795 31853992

[28] Foglio AM McNamara CT Lindeque BG Greyson MA. Technical strategies for harvest of the subtotal pedicled fillet of thigh flap for reconstruction of external hemipelvectomy and hemicorporectomy defects. Plast Reconstr Surg Glob Open. (2023) 11(6):e4993. 10.1097/GOX.0000000000004993 37396841 PMC10313270

[29] Boyd JB Taylor GI Corlett R. The vascular territories of the superior epigastric and the deep inferior epigastric systems. Plast Reconstr Surg. (1984) 73(1):1–16. 10.1097/00006534-198401000-00001 6197716

[30] Wenny R Schmidt M Zaussinger M Zucal I Duscher D Huemer GM. Microvascular free flaps from the lower abdomen for preservation of amputation length in the lower extremity. Clin Hemorheol Microcirc. (2021) 78(3):283–90. 10.3233/CH-211112 33682702

[31] Eric M Ravnik D Zic R Dragnic N Krivokuca D Leksan I. Deep inferior epigastric perforator flap: an anatomical study of the perforators and local vascular differences. Microsurgery. (2012) 32(1):43–9. 10.1002/micr.20944 22113874

[32] Holm C. Perfusion zones of the DIEP flap revisited: a clinical study. Plast Reconstr Surg. (2006) 118(4):1077. 10.1097/01.prs.0000233604.86936.e8 16980874

